# Individual participant data meta-analysis of prognostic factor studies: *state of the art?*

**DOI:** 10.1186/1471-2288-12-56

**Published:** 2012-04-24

**Authors:** Ghada Abo-Zaid, Willi Sauerbrei, Richard D Riley

**Affiliations:** 1 European Centre for Environment and Human Health, Peninsula College of Medicine and Dentistry, University of Exeter Knowledge Spa Royal Cornwall Hospital Truro, Cornwall, TR1 3HD, UK; 2 Institute of Medical Biometry and Medical Informatics, University Medical Center Freiburg, Freiburg, Stefan-Meier-Strasse 26, 79104, Germany; 3 School of Health and Populations Sciences, Public Health Building, University of Birmingham,, Edgbaston, Birmingham, B15 2TT, UK

**Keywords:** Meta-analysis, Prognostic factor, Prognosis, Individual participant (patient) data, Systematic review, Reporting

## Abstract

**Background:**

Prognostic factors are associated with the risk of a subsequent outcome in people with a given disease or health condition. Meta-analysis using individual participant data (IPD), where the raw data are synthesised from multiple studies, has been championed as the gold-standard for synthesising prognostic factor studies. We assessed the feasibility and conduct of this approach.

**Methods:**

A systematic review to identify published IPD meta-analyses of prognostic factors studies, followed by detailed assessment of a random sample of 20 articles published from 2006. Six of these 20 articles were from the IMPACT (International Mission for Prognosis and Analysis of Clinical Trials in traumatic brain injury) collaboration, for which additional information was also used from simultaneously published companion papers.

**Results:**

Forty-eight published IPD meta-analyses of prognostic factors were identified up to March 2009. Only three were published before 2000 but thereafter a median of four articles exist per year, with traumatic brain injury the most active research field. Availability of IPD offered many advantages, such as checking modelling assumptions; analysing variables on their continuous scale with the possibility of assessing for non-linear relationships; and obtaining results adjusted for other variables. However, researchers also faced many challenges, such as large cost and time required to obtain and clean IPD; unavailable IPD for some studies; different sets of prognostic factors in each study; and variability in study methods of measurement. The IMPACT initiative is a leading example, and had generally strong design, methodological and statistical standards. Elsewhere, standards are not always as high and improvements in the conduct of IPD meta-analyses of prognostic factor studies are often needed; in particular, continuous variables are often categorised without reason; publication bias and availability bias are rarely examined; and important methodological details and summary results are often inadequately reported.

**Conclusions:**

IPD meta-analyses of prognostic factors are achievable and offer many advantages, as displayed most expertly by the IMPACT initiative. However such projects face numerous logistical and methodological obstacles, and their conduct and reporting can often be substantially improved.

## Introduction

Prognostic factors are measurable characteristics associated with the risk of a subsequent outcome in people with a given disease or health condition. They include simple measures such as age or body mass index, and more complex measures such as biomarkers and genetic factors. For example, age, glucose levels, motor score and pupillary reactivity are associated with a higher risk of poor outcome in patients with traumatic brain injury [1]. In breast cancer patients, higher levels of markers uPA and PAI-1 are associated with a shorter time to disease recurrence or death [2]. Prognostic factors have a broad array of potential uses in both clinical practice and health research [3]. For instance, they help to define disease at diagnosis (e.g. cancer diagnosis is usually accompanied by the stage of disease, based on the prognostic factors of tumour size, nodal status, and metastasis); they aid the design and analysis of trials [4]; they are confounders to consider in observational studies and unbalanced trials [5]; they are the building blocks of risk prediction models [6]; and they may even predict treatment response [7].

Primary research studies to identify prognostic factors are common place in the health and epidemiology literature. However, meta-analysis of prognostic factor studies has proven problematic, with most attempted synthesises only serving to highlight the poor quality of primary studies and their heterogeneous nature (e.g. in their choice of cut-off levels, method of measurement, analysis strategies etc) [8-10]. Even when good quality studies are available, meta-analysis is usually still halted by difficulties extracting summary data (e.g. hazard ratios and their confidence intervals) for synthesis [8]. For example, Sutcliffe et al.[11] conclude that: 

"“The considerable variability in results reported within the prognostic marker categories, the poor quality of studies and the lack of studies for some categories have made it difficult to provide clear conclusions as to which markers might offer the most potential as prognostic parameters for localised prostate cancer. These reasons also meant that it was not possible to quantitatively synthesise the results’."

**Figure 1:**
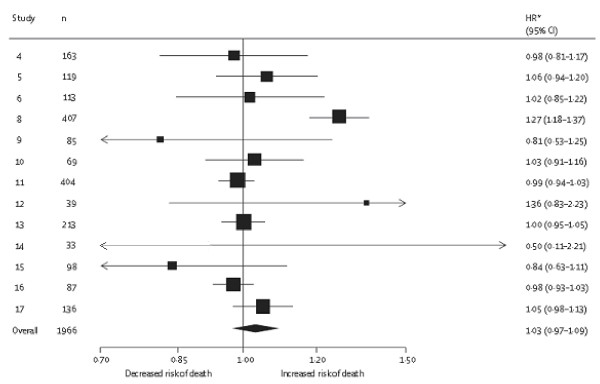
** An IPD meta-analysis of whether microvessel density is a prognostic factor for death in patients with non-metastatic surgically treated non-small-cell lung carcinoma, as undertaken by Trivella et al.**[14]. The forest plot shows the individual study hazard ratio estimates (with confidence intervals), which indicate the association between risk of death and an increase of ten microvessel counts, as assessed by measurement of all vessels. A random-effects meta-analysis was used to combine estimates (I^2^ = 73.7%), and the overall hazard ratio shown is thus the estimated average of all the underlying hazard ratios across studies.

Meta-analysis using individual participant data (IPD) [12], where the raw data are obtained from multiple studies and synthesised, has been championed as a potential solution for synthesising prognostic factor studies [13-15]. IPD is the original source material and thus, in an ideal situation, offers numerous potential advantages including [16]: standardizing statistical analyses in each study; deriving summary prognostic factor results directly, independent of study reporting and significance; checking modelling assumptions; performing adjusted analyses in each study with a consistent set of adjustment factors; examining non-linear associations and interactions between prognostic factors; and explaining heterogeneity in prognostic factor effects (e.g. across subgroups of patients, or across studies with different methodological standards). Altman et al.[17] show in non-small-cell lung carcinoma that an IPD meta-analysis of prognostic factors (IMPF) is achievable, and can reveal important findings. Their meta-analysis of 13 studies is presented in Figure 1[14], and it shows that microvessel density, when measured by counting all vessels and analysed as a continuous variable, is not a prognostic factor for death (summary hazard ratio = 1.03, 95% CI: 0.97 to 1.09), contradicting a previous meta-analysis of published summary data [18].

However, despite these potential advantages, in reality the synthesis of IPD may itself have severe problems [19]. For example, availability of IPD does not overcome poor quality of primary studies; IPD may not be available from every study desired; and studies may differ in the set of prognostic factors (confounders) recorded and their method of measurement. The IPD approach is also known to be potentially costly and time-consuming [20], which dissuades many researchers. For example, the IPD meta-analysis project of Altman et al.[17] was a ‘*long, expensive, and rather laborious process*’.

In this article we undertake a systematic review to identify and then critically appraise published IMPF articles. The aim is to assess the feasibility of the IMPF approach, to examine how it is conducted (e.g. how IPD are obtained, how statistical analyses are performed etc.), to evaluate how IMPF are reported, and to identify common challenges facing IMPF projects. By evaluating published articles, our review findings are clearly dependent on reporting standards within the articles, and so any apparent research deficiencies identified may merely reflect poor reporting standards. However, whilst recognising this limitation, our review findings will help inform those currently embarking upon or contemplating an IMPF, and direct further methodological research within IPD meta-analysis.

## Methods

### Inclusion and exclusion criteria

An IMPF article was defined as one which reported, as a primary objective, a meta-analysis using IPD to assess the prognostic ability of one (or more) factors in patients with a specific disease or health condition. A ‘factor’ was defined as any type of variable that could be measured quantitatively, and so for example could relate to binary, categorical or continuous measures. A ‘meta-analysis using IPD’ was defined as the synthesis of raw patient-level data across multiple studies or multiple collaborating groups. There were no restrictions on the type of prognostic factors, or the disease/health condition under investigation, or the types of study data (e.g. randomised trials, observational studies) being synthesised. Articles focusing on risk factors for disease onset (aetiology) in healthy individuals, and methodological articles focusing on methods for conducting an IMPF were excluded. Articles examining prognostic factors as a secondary objective were also excluded; for example, studies with a primary objective to assess a treatment effect were excluded, even if they adjusted for prognostic factors or looked at how prognostic factors predicted treatment response. Articles with a primary objective to develop a risk prediction model [21] (i.e. a model that predicts individual outcome risk using multiple prognostic factors in combination) were excluded unless a concurrent primary objective was to assess the prognostic ability of the individual factors being considered for model inclusion.

### Search strategy

It was deemed difficult to search for IMPF articles directly, due to the inconsistent nomenclature within prognosis research [22] (e.g. ’predictive’, ’prognostic’; ‘factor’, ‘marker’) and the wide range of potential prognostic factors (e.g. genes, chromosomes, biomarkers, clinical characteristics, etc). Thus, initially a broader search was undertaken for any type of IPD meta-analysis article. An existing database of 199 such articles was already available from a previous systematic review of the health literature from 1996 to 2005 [23]. This database was updated by searching within Medline, Embase and the Cochrane library from the 1st January 2005 to March 2009 using the same search strategy as used before [23]. We also crudely searched Google using ’individual patient data meta-analysis’. The abstracts of all articles identified by the search were read by the first author and classified in regard their IPD meta-analysis status as either ’yes’, ’unsure’, or ’no’. The second author checked all ‘yes’ and ‘unsure’ articles, a random 10% of the ‘no’ articles, and any ‘no’ articles that contained ‘individual patient data’ or ‘individual participant data’. Any discrepancies were resolved by obtaining the full paper.

The abstract of each article classed as an IPD meta-analysis was then read again and further classed by the first author as ‘IMPF yes’, ‘IMPF unsure’, and ‘IMPF no’. The second author checked all these classifications, and any discrepancy resolved by obtaining the full paper. Finally, the references of ‘IMPF yes’ articles were checked to identify any relevant articles previously missed.

### Data extraction and in-depth evaluation of recent IMPF articles

Each article classed as an IMPF was obtained in full and the following information extracted: year of publication, location of first author, disease/health condition of participants, outcomes of interest, and prognostic factors examined. A more in-depth evaluation was then performed of a random sample of 20 articles published in or after 2006. All articles published from 2006 were listed randomly using computer software, and the first 20 in the list chosen. Twenty was considered appropriate for pragmatic reasons (e.g. time) and sufficient for identifying the key issues, limitations and challenges of IMPF projects; we were less interested in getting reliable estimates of the related percentages. A data extraction form was developed that included 58 questions (available on request). The first author answered these questions by eliciting the relevant pieces of information from the full published article; these answers were then checked by either the second or third authors, and any discrepancies resolved. The 58 questions covered the rationale, conduct, analysis, reporting [24,25], and feasibility of the IMPF project (Table 1). These questions were part of the protocol for our review, which is available upon request.

**Table 1:** Summary of the data extraction form involving 58 questions, which was used to extract information about the 20 IMPF projects examined in detail

***Rationale and initiation:***	We recorded the rationale for the IMPF, and whether there was mention of a project protocol and ethics approval.
***Process of obtaining IPD:***	We recorded how researchers identified relevant primary studies (e.g. systematic review, coalition of research groups); how they decided which studies to seek IPD from; the process of obtaining IPD; and problems encountered.
***Details of IPD obtained:***	We recorded the proportion of studies providing IPD; the total number of participants in the IPD; whether the number of participants and events were reported for each IPD study; whether there was any missing data problems; and whether there was variability in how prognostic factors were measured.
***Type and quality of IPD studies:***	We recorded the design (e.g. cohort, randomised trials) of studies providing IPD; whether they were published or unpublished; and whether they were assessed for their quality and, if so, how.
***Statistical methods used:***	We recorded whether a statistical methods section was provided; the statistical models used in the meta-analysis (e.g. Cox regression, logistic regression); and how some specific statistical issues were addressed (such as clustering of participants within studies; between-study heterogeneity in prognostic factor effects; and the analysis of continuous prognostic factors).
***Assessment of publication bias and availability bias:***	We recorded if and how researchers examined the potential impact of publication bias (studies unpublished due to non-significant prognostic results) or availability bias (studies providing IPD are a biased portion of the studies from which IPD was desired) in their meta-analysis.
***Adherence to reporting guidelines:***	As a crude measure of adherence to reporting guidelines for meta-analysis, we recorded how many of the articles referenced the reporting guidelines of either MOOSE [24] or QUORUM [25].
***Limitations and challenges of an IMPF:***	We catalogued all the problems that hindered the IMPF approach as reported by the researchers.

## Results

### Search and classification results

**Figure 2:**
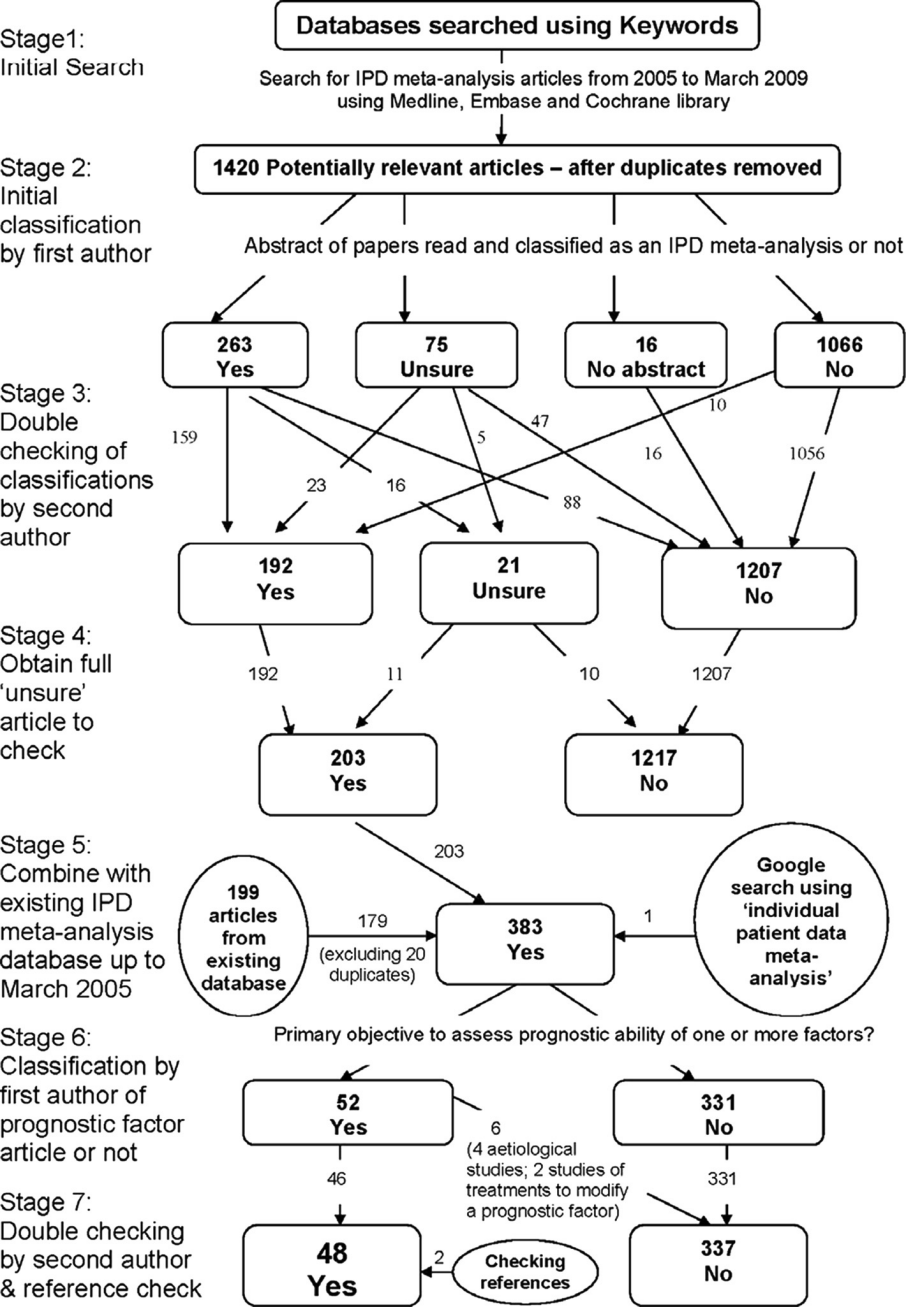
Details of the search and classification of IMPF articles.

We identified 385 general IPD meta-analysis articles published between 1991 and March 2009, including 179 from a previous IPD database plus 204 from our new searches and 2 from reference checking (Figure 2). Of these, we classed 48 as having a primary objective to assess the prognostic ability of one or more factors in patients with a defined disease or health condition. A full list of these references is available upon request.

### Overview of the 48 IMPF articles

The 48 IMPF articles consider prognostics factors in a broad range of diseases and health conditions (Additional file 1: Figure S1). The most active research area is within traumatic brain injury, where numerous IPD meta-analyses have been completed because researchers initiated IMPACT (International Mission for Prognosis and Analysis of Clinical Trials in traumatic brain injury) and shared IPD from 11 studies (8 randomised trials and 3 observational studies) including 9205 patients for examining issues related to heterogeneity between, and prognosis of, patients in clinical trials [26]. Mortality and disease recurrence were the two most common outcomes of interest across the 48 articles, with other outcomes tending to be condition-specific (e.g. Glasgow Outcome Scale score in traumatic brain injury; middle ear effusion in acute otitis media). Age, sex and blood pressure were the most common factors examined for their prognostic ability, again alongside condition-specific factors (e.g. microvessel density counts in lung cancer [14], restrictive mitral filling pattern in cardiovascular disease [27]).

**Figure 3:**
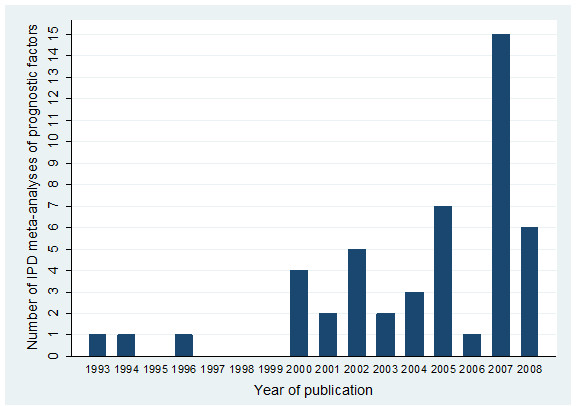
** Number of published IMPF articles over time (NB no articles were identified in 2009 up to the start of March, when our review was conducted); the spike in 2007 is due to eight articles**[28-35]**from the IMPACT collaboration being published simultaneously within the Journal of Neurotrauma.**

Only 3 published IMPF articles were identified before the year 2000 but thereafter a median of 4 articles exist per year (Figure 3). In 2007 there was a peak of 15 IMPF articles, due to 8 IMPACT articles [28-35] being published simultaneously within the Journal of Neurotrauma, with each article generally focusing on a different class of factors (e.g. demographic, laboratory variables, coma scale score, etc).

### In-depth evaluation of 20 recent IMPF articles

Our in-depth evaluation was performed on a random sample of 20 IMPF articles published from 2006 to 2008 [14,27-33,36-47]. These included six articles from the IMPACT collaboration [28-33]; when evaluating these articles, we also utilised information published simultaneously in companion articles describing the IMPACT study, its design, and statistical analysis plan [4,26,48]. We did this to gain broader insight into the IMPACT series as a whole, as clearly quality issues in a single article are dependent on quality issues in the entire IMPACT project. Further, as standards within the six IMPACT articles are related, below we typically note if and how results for the IMPACT project differed to the 14 non-IMPACT articles. We now summarise the key findings in relation to the eight categories defined in Table 1.

#### Rationale and initiation

A common rationale for the IMPF projects was to increase statistical power compared to individual studies alone, and to resolve disagreements in the field; for example, the rationale for Lanterna et al.[45] was that *'emerging evidence suggests that the APOE4 allele may increase the risk of a negative outcome in patients with aneurysmal subarachnoid hemorrhage, but the results are conflicting.'* Another common objective was to identify independent prognostic factors; i.e. those factors with prognostic value even after adjustment for other standard factors. For example, Trivella et al.[14] assess whether microvessel-density has prognostic value in non-small-cell lung carcinoma when adjusting for other variables including age and stage of disease (Figure 1). In six of the 20 articles, a concurrent objective was to develop a risk prediction model after the prognostic factors were identified. The risk prediction model component (e.g. development [6], application [21], validation [14]) of these articles is beyond the scope of our evaluations here. Of the 20 articles, fifteen (including the 6 IMPACT articles) mentioned that the project was funded. Only 3 articles[27,40,46] directly stated there was a protocol for the project, and only 1 mentioned they had ethics approval[27]. The IMPACT articles did not mention ethics approval or a protocol, although the latter can perhaps be inferred by the existence of separate articles describing the objectives, rationale and analysis plan for the IMPACT initiative.

#### Process of obtaining IPD

Nine of the 20 IMPF articles used a literature review to identify primary studies for which IPD was desired [14,27,37,38,40,43-46]. In the 11 others: the six IMPACT articles utilised those studies already providing IPD within the IMPACT database directly [28-33]; one utilised a set of known German trials [42]; one contacted colleagues in the field who had directed relevant trials with placebo arms [39]; one identified studies from a recent systematic review and ‘from our files’ [47]; and two did not state how they identified relevant studies [36,41].

Six of the nine articles using a literature review approach reported the keywords used for searching, and the most common databases searched were PubMed, Medline, Embase, and the Cochrane databases. Once relevant studies were identified, four of the nine articles explicitly stated how authors were approached for their IPD (three by e-mail and one by letter), four simply said authors ‘were asked’, and one did not mention anything in this regard. Only one of the nine articles provided a flowchart detailing the process of searching, classifying, and retrieving IPD studies [37].

Of the 20 articles, eight revealed some resource related issues for obtaining and managing the IPD, including the six IMPACT articles. Thakkinstian et al. [37] state that data cleaning and checking were performed separately for each study, whilst more strikingly Trivella et al.[14] state that ‘*checking, validation and standardisation of all datasets took nearly two years’* and ‘*for all but three centres some data corrections were necessary*.’ The complexities involved in obtaining and managing IPD in the IMPACT database are thoroughly described by Marmarou et al.[26] who state: ‘the overall process was enormously labour intensive, and required considerable clinical insight’.

#### Details of IPD obtained

**Figure 4:**
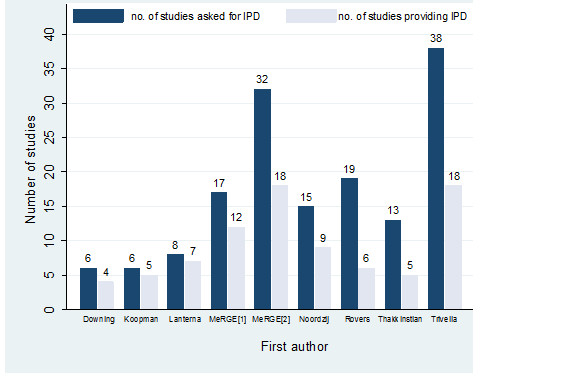
The number of studies for which IPD was requested and obtained in each of the nine IMPF articles using a literature review to identify relevant studies.

All of the nine IMPF articles using a literature review to identify relevant studies did not obtain IPD from all studies desired (Figure 3). Six of these documented why some IPD was unavailable; reasons included non-response to e-mails, IPD no longer available [46], and lack of resources to participate [14]. The percentage of studies providing IPD ranged from 32% to 88%, and five of the nine articles obtained IPD from 60% or less of the requested studies. The shortfall appeared larger in those IMPF articles requesting IPD from 10 or more studies (Figure 4).

All of the 20 articles reported the number of patients included in their available IPD; this ranged from 131[42] to 8721[33], with a mean and median of 3762 and 2954 respectively. Sixteen of the 20 articles (including five of the six IMPACT articles) also reported the number of patients separately for each included IPD study, but only 4 articles reported the number of outcome events separately for each IPD study [36,39,45,47]. Though the number of events was available for each study in the IMPACT database as a whole [26], the six IMPACT articles each used a subset of the available data for which the number of events was not stated.

Missing data was a major problem within the IPD obtained both at the patient-level and at the study-level. All 20 IMPF articles reported one or more of: missing values of prognostic factors and adjustment factors for some patients within a study (e.g. Rovers et al.[43]); missing outcome data for some patients within a study (e.g. Lanterna et al.[45]); and some factors missing completely in some studies (e.g. Trivella et al.[14]).

#### Type and quality of IPD studies

The specific design of included IPD studies was well-documented in the 20 IMPF articles. For example, IMPACT articles utilised IPD from three observational studies and eight randomised trials, from which they included patients in both placebo and treatment arms because: ‘as no trial showed a significant difference between placebo and drug, it was felt that pooling the data would be appropriate for almost all of the analyses which are planned to be undertaken.’ In contrast, some authors (for example Yap et al.[36]) utilised IPD from just the placebo arm of randomised trials, because the prognosis of patients in the treatment arm were not of interest and/or they did not want to model the treatment effect. For example, Koopman et al. [38] state: ‘To eliminate possible effects of antibiotic therapy on the findings, we only included children from the control groups in this prognostic analysis’. Only 2 of the 20 articles clearly mentioned that some of their IPD studies were unpublished [14,27]; neither of these was an IMPACT article.

Eight of the 20 articles reported a quality assessment [49] (or risk of bias [50]) of the studies actually providing IPD. These included the six IMPACT articles, for which Marmarou et al.[26] conclude ‘the data within IMPACT is of as high a standard as can realistically be achieved, as it has been subjected to intense scrutiny by groups funded to provide a high level of quality control’. Thakkinstian et al.[37] examined the Hardy-Weinberg equilibrium in each study, and excluded from the subsequent meta-analysis any study that failed this. Downing et al.[46] state using quality criteria proposed by McKibbon [51] and Altman [14], and they note that all four of their IPD studies had methodological shortcomings. The remaining articles may have assessed study quality *prior* to selecting those studies to request IPD from, but we did not evaluate this.

#### Statistical methods used

All 20 articles provided a statistical analysis description in their Methods section.

##### Statistical models for the patient-level data

To analyse the patient-level data, the most common statistical models used were Cox regression (6 articles), and either logistic or proportional odds regression (11 articles, including all 6 IMPACT articles). Nine of the 20 articles (including all six IMPACT articles) reported checking model assumptions, in regard either the proportional hazards assumption in Cox regression or the proportional odds assumption in odds regression. In eight [27,46] of these nine articles, it was explicitly stated that model assumptions were checked separately in each study included in the meta-analysis; for example, McHugh et al.[48] illustrate how the IMPACT articles checked the proportional odds assumption in individual studies, although they do not report whether this assumption was met in each study and do not say how meta-analysis proceeded if the assumption failed in some studies.

##### Meta-analysis framework

For meta-analysis, nine articles (including the six IMPACT articles) used only a two-step approach where the IPD was firstly analysed in each study separately, and then in the second step the summary data obtained (e.g. hazard ratios, odds ratios) were synthesised using a traditional model for meta-analysis of aggregate data [52,53]. Ten articles used a one-step approach, where the IPD across all studies were analysed together simultaneously. One article used a one-step approach in some analyses and a two-step approach in others.

Those articles using a two-step meta-analysis framework naturally account for the clustering of patients within studies by analysing each study separately in the first step. However, five of the 11 articles using a one-step method did not state they adjusted for clustering of patients within studies; the one-step method requires specific adjustment for study (e.g. by including a dummy variable for each study) otherwise it treats the IPD as if all coming from a single study.

##### Assessing and accounting for heterogeneity in prognostic factor effects

Between-study heterogeneity in prognostic effects (e.g. hazard ratios, odds ratios) was examined in 16 of the 20 articles (including all six IMPACT articles), typically using the I^2^ statistic [54] or the Q-statistic (chi-square test for heterogeneity) [55]. Of the 20 articles, ten (including the six IMPACT articles) specifically accounted for between-study heterogeneity in their meta-analysis model by including random-effects; seven did not account for heterogeneity but justified why, for example as it was negligible (e.g. Koopman et al.[38] note small values of I^2^ < 25%) or the chi-square test was non-significant (e.g. Thakkinstian et al.[37] note the test gave p > 0.1); two articles did not account for heterogeneity in their meta-analysis but did not explain why; and in the remaining article heterogeneity was examined but it was unclear whether it was accounted for in the meta-analysis. Three of the 20 articles [14,45,47] examined potential causes of between-study heterogeneity; for example, Trivella et al. [14] perform subgroup analyses according to the method of measuring microvessel density.

##### Analysis of continuous factors

Nineteen of the 20 articles (including all six IMPACT articles) investigated one or more factors measured on a continuous scale. Of these 19, eight (including one of the six IMPACT articles) converted the continuous factors to a categorised scale for the analysis; five (including two IMPACT articles) analysed the continuous factors on a continuous scale; and six (including three IMPACT articles) used a continuous scale in some analyses (or for some factors) and a categorised scale in other analyses (or for other factors). Note that we did not examine if the handling of continuous factors differed according to whether they were of primary interest or secondary interest (e.g. as confounders).

Six of the 11 articles using a continuous scale (including four of the five IMPACT articles that used a continuous scale) modelled non-linear trends by using either a spline function or polynomial terms, and one other article (an IMPACT article) stated linear trends were most appropriate. Further, in seven of the 11 articles maintaining a continuous scale, the original continuous scale was changed for the analysis. For example Goetz et al. [39] divide age in years by 10, to give the prognostic effect of a 10 year increase in age, whilst Yap et al.[36] log-transform skewed continuous variables. To aid understanding after non-linear trends were fitted, the IMPACT articles often reported the results by comparing groups defined by the 25^th^ and 75^th^ percentile of the continuous variable. Further, to produce more interpretable effect estimates, the IMPACT articles also divided age by 10 years.

Eleven of the 14 articles that used a categorisation justified their choice of categories. For example, any categorisation within an IMPACT article was done after inspecting fitted spline functions for a U-shaped relationship, then defining three categories that described this. In contrast, in another article the choice of categorisation appeared due to statistical significance [42]: *‘Age and percentage of +8 positive metaphases were included dichotomized as cut-points were found in the hierarchical cluster analysis at 45 years (p = 0.001) and 80% (p = 0.04), respectively.’* The remaining articles stated choosing categories to make results clinically meaningful, or choosing cut-points to mirror those used in previous reports, or having to use cut-off points imposed on them by the original studies. For example, Rovers et al. [43] note that ‘*some predictor and outcome variables (e.g. fever and pain) might have been more informative if analyzed on a continuous scale. Some trials did measure these items on a continuous scale but, because others did not, we needed to recode these items as dichotomous variables*.’ Obviously, summary meta-analysis estimates for prognostic factor categories defined as ‘low values’ with ‘high values’ (or ‘low’, ‘middle’, ‘high’) are hard to interpret if different cutpoints are used in the individual studies to define what is a ‘low’ or a ‘high’ value.

##### Assessment of independent prognostic value

Of the 20 articles, 16 (including all 6 IMPACT articles) reported a multivariable analysis to examine the independent prognostic value of one or more factors after adjusting for others. Of these 16, seven (including one IMPACT article) defined the statistical significance criteria by which they judged a factor to have independent prognostic value. For instance, in Koopman et al. [38] a criteria of p < 0.05 was used for statistical significance in the multivariable model and thus evidence of independent prognostic value. Eleven of the 16 articles (including all six IMPACT articles) reported the results in full (i.e. adjusted effect estimate with uncertainty or p-value) for those factors deemed not to have independent prognostic value.

##### Assessment of interactions between prognostic factors

IPD offers the opportunity to examine whether the interaction between two or more factors is itself prognostic. This is rarely considered in primary studies, but IPD from multiple studies offers improved power for such assessments. Seven of the 16 articles (including two of the six IMPACT articles) considered a possible interaction between two or more factors in their multivariable model. For example, in their IMPACT article McHugh et al. [33] considered whether the interaction between hypoxia and hypotension is prognostic for 6-month outcome in patients with traumatic brain injury.

#### Assessment of potential biases

##### Publication bias

Of the 20 articles, only 2 articles (both non IMPACT articles) undertook a formal assessment of whether publication bias or small study effects (i.e. the tendency for small studies in the meta-analysis to give more favourable prognostic effects than the larger studies) may be affecting their meta-analysis. Lanterna et al.[45] assessed the presence of publication bias using a funnel plot (odds ratios on the *x* axis and trial size on the *y* axis) and Egger’s test for asymmetry [56]. In the other article [40] the authors state: ‘*The present result may be subject to publication bias. However, inspection of the funnel plot of the individual hazard ratios for each included study failed to identify important heterogeneity (I*^*2*^*= 0.042, P = 0.06)’*; their choice of method here confuses us, as presence of heterogeneity does not imply funnel plot asymmetry or publication bias.

##### Availability bias

The six IMPACT articles [28-33] utilised IPD from 11 studies, including eight trials. However, Maas et al. [4] list 21 relevant trials in the field, and so 13 trials did not provide their IPD, either because investigators refused to collaborate (potentially because their trial was not yet published) or because IPD were no longer retrievable. Despite this, the threat of bias in the IPD available for the IMPACT database was considered low, as Marmarou et al.[26] note that ‘part of the requirements for ABIC (the American Brain Injury Consortium) and EBIC (the European Brain Injury Consortium) when taking on a TBI study is that the data should ultimately be made available for use in academic projects such as IMPACT.’ Furthermore, the provision of a trial’s IPD was unlikely to be associated with the significance of its prognostic factor results, as the assessment of prognostic factors was not the original aim of any trial.

Of the remaining 14 articles: two obtained IPD from all desired studies, two did not report their success rate, and 10 did not obtain IPD from all desired studies. Four [27,40,45,46] of these latter ten articles reported the number of patients in the non-IPD studies, but only one reported the number of events in the non-IPD studies. Only three [37,43,46] of the 10 articles considered the robustness of their conclusions to the inclusion/exclusion of the non-IPD studies (availability bias). For example, Rovers et al. [43] state ‘*6 of the 10 relevant randomized trials were included in our meta-analysis. The main-effect results for the 4 trials whose individual patient data were not available were very similar to those for the included trials … therefore, it is not expected that inclusion of these data would have changed the results of this meta-analysis.’* However, similarity in main effects of treatment comparisons does not imply that the effects of prognostic factors are not affected. In Thakkinstian et al. [37] the authors use reported summary data in the non-IPD studies to partially reconstruct the unavailable IPD [23,57]; they then combine the fully available IPD with this reconstructed IPD in logistic regression models, and show that findings remain statistically non-significant.

#### Adherence to reporting guidelines

Only two [27,40] of the 20 articles mentioned using reporting guidelines, with both referring to the MOOSE guidelines [24].

#### Limitations and challenges of an IMPF

We elicited numerous challenges of an IMPF that were mentioned in one or more of the 20 IMPF articles. These are summarised in Table 2, and include issues with identifying relevant studies and obtaining IPD; dealing with specific (analysis) issues within the individual studies; dealing with heterogeneity across studies; and the choice of meta-analysis method. The three most common problems were unavailability of IPD for some studies, missing data, and different methods of measuring and recording (prognostic) factors across studies. Eighteen of the 20 IMPF articles explained how missing data was handled, which included one or more of : restricting analyses to those participants with complete data for prognostic factor and outcome; assessing only those factors recorded in every study; and imputation of missing participant-level values for factors and outcome using a statistical method such as multiple imputation. The IMPACT articles often imputed any missing outcomes and missing prognostic factors using an imputation approach.

**Table 2:** Challenges facing researchers conducting an IMPF

**Identifying all relevant studies**	
·	Unavailability of IPD in some studies
·	Time-consuming and costly nature of obtaining, cleaning and analysing the IPD.
**Issues within individual studies**	
·	Dealing with skewed continuous variables and possible outliers.
·	Inability of IPD to overcome deficiencies of original studies, such as being retrospective rather than prospective, being too small for a multivariable analysis, missing important confounders, missing participant data or being of low methodological quality, etc.
·	How to assess the quality of studies identified
·	Re-analysing individual study IPD before considering meta-analysis. For a summary of important issues for the analysis of single prognostic factor studies see Holländer and Sauerbrei [9]. The re-analysis of a single study as the preliminary or first step toward a meta-analysis is influenced by and has consequences for the meta-analysis strategy (15).
**Heterogeneity between studies**	
·	Different definitions of disease or outcome; e.g. Noordzij et al.[44] note different definitions of hypocalcemia across studies, whilst the MeRGE [40] collaborators note the definition of acute myocardial infarction changed over time.
·	Different participant inclusion and exclusion criteria
·	Different methods of measuring the same prognostic factor, for example see difficulties described by Look et al [2].
·	For survival data different lengths of follow-up
·	Factors measured at different points in time or at different stages of disease across studies; e.g. the MeRGE [40] collaborators note that the timing of echocardiography was variable in their included studies, although within 2 weeks of the index acute myocardial infarction
·	Different (or out-dated) treatments strategies, especially when a mixture of older and newer studies are combined; e.g. Yap et al. [36] state that a large proportion of the patients in their included trials did not receive common post-myocardial infarction therapy such as β-blockers and ACE inhibitor.
·	Insufficent information about treatment for some of the studies.
**Statistical issues for meta-analysis**	
·	Missing data, including: missing factor values and outcome data for some participants within a study, and unavailable factors in some studies
·	Inability to adjust prognostic effects for a consistent set of adjustment factors in each study
·	Different measurement techniques between studies may be acceptable for adjustment variables, but are critical for the variable of main interest
·	Insufficient information to separate patient outcomes more discretely, e.g. Thakkinstian et al. [37] could not separate chronic allograft nephropathy from graft rejection or acute rejection from chronic rejection
·	Imposed choice of cut-off levels when individual studies categorise their continuous variables and/or categorise their continuous outcomes in their provided IPD
·	Difficulty in using a continuous scale for continuous factors in meta-analysis when some studies give IPD values on a continuous cale and others do not (e.g. see Rovers et al. [43])
·	Considering whether it is sensible and/or possible to investigate differential prognostic effects in subgroups
·	Potential for study-level confounding when assessing whether study covariates (e.g. year of publication) modify the prognostic effect.
·	Difficulty of interpreting summary meta-analysis results in the presence of heterogeneity (and heterogeneous populations) across studies.
**Assessment of potential biases**	
·	Potential for publication bias and availability bias
·	How to assess the robustness of IPD meta-analysis results to the inclusion/exclusion of studies only providing summary data; and how to combine IPD studies with summary data studies

For the main factors of interest, seven articles had the different methods of measurement problem , and three of these tried to limit this. For example, Trivella et al. [14] perform a subgroup analyses for each of two methods (Chalkley vs all vessels) used to count the factor microvessel density. However, they note that even when the same counting method was used to count microvessel density, individual laboratories still used very different procedures for measurement of microvessel density. The other four articles did not attempt to tackle the problem but still presented summary results that thus represented some average prognostic effect across all measurement scales. One of these, Thakkinstian et al.[37], notes this limitation: *‘since the IPD meta-analysis is a retrospective collaboration, it is difficult to get clinical variables that have been assessed and measured using similar methods across all studies; standardization is best done as a prospective collaboration.’*

## Discussion

Our review found 48 IMPF articles, which shows an IPD synthesis of prognostic factors is achievable, and the approach has become more popular since the year 2000.

This should encourage researchers interested in the IPD approach, such as Broeze et al.[58] who raise awareness of the benefits of IPD but state that ‘to our knowledge, no IPD meta-analyses of diagnostic or prognostic research have been conducted so far’.

The IMPACT initiative is a leading light in the field, contributing eight of the 48 articles with generally strong design, methodological and statistical standards throughout; it should be a first calling point for researchers wishing to undertake a similar project. The IMPACT and other IPD projects identified reflect researchers’ increasing awareness that a meta-analysis of aggregate data from observational studies is problematic and unreliable [59], as well documented for some time in the epidemiological literature [15,60]. However, our in-depth assessment of 20 recent IMPF articles identified that the IPD approach itself has many challenges, with numerous logistical and methodological issues to consider, as summarised in Table 2. There are also areas where the conduct and reporting of IMPF projects can be improved. Obviously, for prognostic factors it is a long way from single studies to an evidence based assessment in meta-analyses [61]. The key findings of our review, and its limitations, are now discussed further, and in Table 3 we outline some key issues for researchers to consider when planning and undertaking an IMPF. 

**Table 3:** Important considerations for those planning and undertaking an IMPF project

**Rationale & Initiation**	
·	Produce a protocol for the IMPF project prior to its initiation (detailing all aspects of rationale, conduct and statistical analysis) and reference this upon publication of the IMPF
·	Consider whether ethics approval is necessary for the IMPF project, and report this upon publication
**Process of obtaining IPD**	
·	Report how primary study authors were approached to obtain their IPD
·	Report the strategy used for searching the literature for relevant studies (if relevant), including keywords used and databases searched.
·	Provide a flowchart showing the search strategy, classification of identified articles, and retrieval of IPD from relevant studies (where relevant)
·	Consider how to improve retrieval of IPD from unpublished studies
**Details of IPD obtained**	
·	Report number of participants and events for each included study
·	Report a summary of the missing data for each study
·	Report the reasons why IPD was unavailable for some studies (if relevant), and if possible, report the number of participants, number of events and summary prognostic factor results in such studies
**Type and quality of IPD obtained**	
·	Consider and report the quality of studies for which IPD were obtained; in particular, are they all of comparable quality?
**Statistical methods used**	
·	Check and report the assumptions of the statistical models used; in particular, do model assumptions appear valid in each study separately?
·	Where possible, analyse continuous factors on their continuous scale and consider non-linear trends. Univariate analyses are a good starting point, but a multivariable analysis adjusting for ‘standard’ factors is required to assess the added prognostic value of a factor over ‘established’ factors.
·	In multivariable analyses consider carefully which variables can and should be used for adjustment. Sensitivity analyses should be conducted. In a similar way consider how treatment differences can be handled in the analysis.
·	In multivariable analyses, define the criteria used to decide whether a factor has independent prognostic value over other factors; also potentially consider whether the interaction between two (or more) prognostic factors is important
·	Consider a re-analysis of the IPD in each study as a first or preliminary step toward meta-analysis, to better appreciate the issues within each study first.
·	In the meta-analysis, account for clustering of participants within studies (and do not merge IPD and analyse as if IPD all came from a single study) and report how this was done.
·	Measure and, if necessary, account for between study heterogeneity in the prognostic factor effect(s) of interest when undertaking meta-analysis
·	Where sufficient studies are available (e.g. 10 per covariate of interest) and heterogeneity of estimated effects of interest exists, examine the potential causes of such heterogeneity.
·	Consider a sensitivity analysis to assess whether meta-analysis conclusions change when restricting to IPD from the higher quality studies (if relevant)
**Assessment of publication bias and availability bias**	
·	Consider the potential impact of publication bias and availability bias on IPD meta-analysis results; in particular, are studies providing IPD comparable to those studies not providing IPD (if relevant)?
**Reporting guidelines**	
·	Utilise reporting guidelines for meta-analysis, such as those for MOOSE [24] and IPD meta-analysis [16]

### Limitations of our review

To identify IMPF articles we used a systematic review that involved a broad search strategy to identify general IPD meta-analysis articles, followed by their classification as an IMPF or not. Although we searched four of the largest electronic databases for relevant articles, other databases do exist, and so it is possible some additional IMPF articles may exist. We also only checked about 10% of those articles classed as ‘not an IPD meta-analysis’ by the first author, although Figure 1 shows that the first author’s initial classifications tended to be cautious (i.e. overly inclusive rather than exclusive). However, we believe that the main messages from our review (that IMPF projects are achievable but face many methodological and practical challenges) are unlikely to be altered by any missing IMPF articles, or that only 20 of the 48 articles were evaluated in full.

Another limitation is that the information extracted from the 20 recent IMPF articles is dependent on reporting standards therein, and so apparent deficiencies within an IMPF project may be confounded by poor reporting. For example, only 3 IMPF articles referred to a protocol for their project, but this does not necessarily mean a protocol did not exist in the other 17 articles. Thus apparent gaps in study conduct may simply relate to gaps in study reporting. This is particular important given we only elicited information directly available in the published IMPF article, and did not utilise other reports (e.g. protocols, statistical analysis plan) or contact authors for information directly.

### How should an IMPF be initiated?

Our review revealed two competing approaches to initiating an IMPF: perform a (systematic) literature review and seek IPD from relevant studies identified, or set-up a collaborative group of selected researchers who agree to share their IPD. It is difficult to recommend one approach over the other, and the main aim – for example, to investigate one or two specific factors, or to investigate many factors simultaneously as in IMPACT - also has an important influence on possible approaches. The systematic review approach is potentially more thorough, as it seeks to identify and utilise all existing evidence, but it is also more resource intensive [14] as large effort is required to identify relevant studies, liaise with study authors, and then obtain and clean IPD provided. Further, at the end of the process IPD is often not obtained from all identified studies anyway (Figure 4). The collaborative group approach is thus appealing, as it is quicker and uses IPD immediately available from collaborators, but one concern is that studies within the collaboration may be a biased selection of the existing evidence (see below). It is thus important for collaborative groups to be ‘well-defined’ or inclusive, and not select collaborators (or datasets) based on significance of their prognostic factor results. There are at least two possible strategies to help overcome this dilemma. The first is a collaboratively pre-planned IPD meta-analysis as discussed in the context of observational studies in epidemiology over a decade ago [15]. The second is to seek IPD from only a ‘well-defined’ list of studies, for example only those containing patients in a particular country of interest, or those using the same method of measurement for a particular factor.

Trivella et al. [14] have a good framework that utilises both a literature review and a collaborative group approach; they state that they *‘produced a list of potential participants—i.e. individuals and centres worldwide who were researching lung cancer—by doing a thorough literature search and by getting in touch with professional contacts of members of the steering committee to obtain details of unpublished studies’.* This approach ultimately led to 18 studies providing IPD, the joint largest number of studies in any of the IMPF projects examined, though at the expense of two years to collate, manage, and clean the IPD obtained [16].

### How can IMPF projects be improved?

Many aspects of the conduct and reporting of IMPF projects are done well. For example, all of the 20 articles we assessed provided a statistical methods section in their Methods; all 20 reported the total number of participants within their IPD; 15 of the 20 accounted for heterogeneity in prognostic factor effects or justified why not; and 16 of the 20 considered the independent prognostic importance using a multivariable model. However, the available data from individual studies impose several challenges to derive a sensible summary estimate from an IMPF (Table 2) and we also indentified a number of ways IMPF projects can be improved. Table 3 summarises important considerations when conducting and reporting IMPF projects.

In particular, it was a surprise that protocols and ethics approval for IMPF projects were rarely mentioned. Protocols are essential components of any research project and enhance its credibility, so they should be made and referred to. In terms of ethics, researchers may have not considered this to be relevant if IPD was being used for the same objectives as in the original studies, for which ethical approval may exist. For an IMPF project it will be difficult or even impossible to do more than collecting and reporting the situation in each study. The chairperson from the original study may be asked to contact her or his ethics committee for approval to provide IPD data.

Reporting standards in general must be improved within IMPF. Basic information was often missing, such the number of participants and events within each study providing IPD, and the keywords used to search for relevant studies. Researchers are encouraged to consider recent guidelines for reporting an IPD meta-analysis [16], which supplement existing reporting guidelines for meta-analysis of a non-IPD approach [24,62]. Of course, an improved reporting of primary studies according to the REMARK guidelines [63] is also needed, and this would be most helpful for several steps toward the IMPF project (e.g. examining study relevance according to inclusion/exclusion criteria; identifying outcomes considered and their specific definition; details about available variables and measurement techniques).

In terms of statistical analysis, some IMPF projects chose to analyse continuous factors on a categorised scale without good reason. This approach has severe disadvantages as it loses statistical power and weakens the ability to assess non-linear prognostic factors effects [5]. When continuous factors are presented categorised within the available IPD itself, we question whether a summary meta-analysis result is even sensible if different cutpoints are used across studies.[59] Another issue is that in five articles the meta-analysis method did not appear to account for clustering of participants within studies, and thus treated the IPD as if all coming from a single study, which is not appropriate. Further, four articles did not consider potential between-study heterogeneity in prognostic factor effects, even though heterogeneity is one of the most pertinent considerations in any meta-analysis. Researchers should refer to statistical articles describing how to undertake IPD meta-analyses that account for clustering and heterogeneity, for example [53,57,64-66], and to articles describing how analyse prognostic factors, such as [5,59,61,67].

Another critical issue for is properly acknowledging the heterogeneity of patient populations, treatment of the patients, and the available ‘standard’ prognostic variables used to adjust the effect of the factor of interest in each study. Issues of the heterogeneity concerning patient populations and treatment may be tackled by excluding subgroups of the patients from some of the single studies, by stratifying analyses or by restricting analyses to more homogeneous subgroups. Ideally the same adjustment variables should be used in each study, but one will be restricted by those available in the IPD provided and sometimes relevant factors may be missing, which should be clearly noted. For example, Thakkinstian et al.[37] note that they ‘*could adjust for only a few clinical factors … Other factors (e.g. immunosuppressive drugs and dosage, viral hepatitis infection, duration of dialysis, etc.) that were previously associated with poor outcomes after renal transplantation were not available in the datasets obtained.’*

Where between-study heterogeneity in a prognostic factor’s effect is identified in the meta-analysis, researchers should consider reporting a prediction interval for the potential effect in an individual setting [68]. Currently, following a random-effects meta-analysis, researchers usually focus on the average prognostic effect estimate and its confidence interval. However, due to the heterogeneity, the prognostic effect may be very different to the average effect in a particular population, and this can be quantified by the prediction interval [69].

Perhaps most importantly, publication bias and availability bias were rarely considered in the 20 IMPF articles examined. These problems are crucial to consider, as they may cause the IPD available to be a biased (non-random) portion of a potential prognostic factor’s evidence base [13,19,70]. Publication bias is a well-known concern in meta-analysis, and is likely to be an even greater problem for prognosis studies than for therapeutic trials [22]. In the past prognosis studies have hardly ever been registered, do not have a protocol or pre-defined analysis plan, and generally have poor reporting standards, which all increase the threat of negative findings remaining unpublished [8,10,71]. Having IPD from prognostic factor studies does not negate the fact that additional *unknown*, unpublished studies may also exist [19,70]. Funnel plots and tests for asymmetry (‘small-study effects’) are useful tools for identifying potential publication bias [72], but these were only used in two of the 20 articles. We note that publication bias is unlikely to occur in examples such as IMPACT, where studies were selected based on the availability of particular factors and relevant endpoints, rather than on reported significance of any prognostic factors. In particular, the IMPACT articles included IPD from eight randomised trials, for which prognostic factors were recorded but not of primary interest to the original researchers, and so the request and provision of IPD was independent to knowledge of prognostic factor effects.

Availability bias is an added concern for IPD meta-analysis, and relates to when not all *known* studies provide their IPD [70]. For example, IPD may be less obtainable from smaller studies and/or studies with non-significant findings as they are more likely to have lost or destroyed their IPD. In such situations, comparison of the summary results in IPD and non-IPD studies is useful to see if they are similar, as done by Rovers et al.[43] and Thakkinstian et al.[37].

## Conclusion

In this article we have found that IPD meta-analyses of prognostic factor studies are achievable and, by using a sample of recent articles, we have examined how they are initiated, conducted, analysed and reported. Our findings reveal how current standards can be improved; show that the IMPACT initiative is a leading example for others to follow; expose the potential logistical and methodological challenges facing IMPF projects (Table 2); and identify important considerations for ongoing and future IMPF projects (Table 3). Although new methodological research may help limit some of these problems, a prospectively planned IPD meta-analysis – where researchers agree at the onset of their studies to facilitate an IPD meta-analysis – is the ideal way forward [15]. We agree with McShane et al.[73] who state that for prognostic factor research ‘the necessity of large, definitive prospective studies or prospectively planned meta-analyses … must be recognized.’

## Competing interests

The authors declare that they have no competing interests

## Authors’ contributions

RR conceived and supervised the project. All authors generated the list of questions for evaluating relevant articles. GA-Z classified all articles and performed data extraction; RR checked classifications and data extractions; WS checked data extractions. GA-Z and RR drafted the first version of the manuscript, and WS commented on this version, with all authors helping to revise the article accordingly and generate the recommendations. All authors read and approved the final manuscript.

## Pre-publication history

The pre-publication history for this paper can be accessed here:

http://www.biomedcentral.com/1471-2288/12/56/prepub

## Supplementary Material

Additional file 1 **Figure S1.** Diseases and health conditions of interest in the 48 IMPF articles identified. Click here for file
