# Supplementary material for: Individual participant data meta-analysis of prognostic factor studies: state of the art?
Source: BMC Med Res Methodol. 2012 Apr 24;12:56. doi: 10.1186/1471-2288-12-56 (PMC3413577; doi:10.1186/1471-2288-12-56)
Supplement: Additional file 1 — Figure S1. Diseases and health conditions of interest in the 48 IMPF articles identified. [file 1471-2288-12-56-S1.doc]

**Additional Material:** **Figure A1.** Diseases and health conditions of interest in the 48 IMPF articles identified
